# Decreased 25-Hydroxy Vitamin D Level Is Associated with All-Cause Mortality in Patients with Type 2 Diabetes at High Cardiovascular Risk

**DOI:** 10.3390/metabo13080887

**Published:** 2023-07-27

**Authors:** Alena Stančáková Yaluri, Ivan Tkáč, Katarína Tokarčíková, Zuzana Kozelová, Mária Rašiová, Martin Javorský, Miriam Kozárová

**Affiliations:** 1 Department of Internal Medicine 4, Faculty of Medicine, P. J. Šafárik University and L. Pasteur University Hospital, 04190 Košice, Slovakia; alena.yaluri@upjs.sk (A.S.Y.); katarina.demkova@upjs.sk (K.T.); zuzana.malachovska@upjs.sk (Z.K.); martin.javorsky@upjs.sk (M.J.); 2 Department of Angiology, Faculty of Medicine, P. J. Šafárik University and East Slovak Institute of Cardiovascular Disease, 04011 Košice, Slovakia; maria.rasiova@upjs.sk

**Keywords:** vitamin D, biomarkers, type 2 diabetes, atherosclerosis, all-cause mortality

## Abstract

Cardiovascular diseases are among the leading causes of morbidity and mortality, particularly in individuals with type 2 diabetes. There is a need for new biomarkers to improve the prediction of cardiovascular events and overall mortality. We investigated the association of selected atherosclerosis related biomarkers, specifically osteoprotegerin (OPG), 25-hydroxy-vitamin D (25(OH)D), C-reactive protein (CRP), lipopolysaccharide-binding protein (LBP), and asymmetric dimethylarginine (ADMA), with the occurrence of any cardiovascular event or all-cause mortality (primary outcome) during a 5.6-year follow-up of 190 patients with type 2 diabetes. Data were analyzed using logistic regression to adjust for baseline cardiovascular status and cardiovascular risk factors. The primary outcome occurred in 89 participants (46.8%) during the study. When analyzed individually, 25(OH)D, CRP, and LBP significantly predicted the primary outcome in multivariable models. However, in a model that included all biomarkers, only a decreased level of 25(OH)D remained a significant predictor of the primary outcome. Moreover, the level of 25(OH)D significantly predicted all-cause mortality: a reduction of 10 ng/mL was associated with a two-fold increase in all-cause mortality. Our study thus demonstrates that vitamin D deficiency was the strongest factor associated with the primary outcome and all-cause mortality after a 5.6-year follow-up in patients with type 2 diabetes at high cardiovascular risk.

## 1. Introduction

Atherosclerosis is a multifactorial process affecting the arterial wall and leading to the development of cardiovascular disease (CVD). CVD belongs to major causes of morbidity and mortality worldwide. Multiple well-known factors play a role in the pathogenesis of atherosclerosis, such as dyslipidemia, hypertension, obesity, smoking, and type 2 diabetes (T2D) [1].

T2D is associated with accelerated atherosclerosis leading to 2–3 times increased incidence of CVD in individuals with type 2 diabetes [2]. Several factors have been proposed to explain acceleration of atherosclerosis in diabetes, including increased atherogenic LDL particles, hyperglycemia, hypertension, oxidative stress, and increased inflammation. The main CVD manifestations in patients with T2D are coronary artery disease, heart failure, stroke, and peripheral artery disease which may cause death in about 50% of patients [3]. Therefore, early prevention, diagnosis, and treatment of CVD in T2D is of high importance.

With the development of metabolomics, an intensive search for early metabolic predictors of cardiovascular complications has been ongoing. Inflammatory markers have been extensively studied because of hypothesized important role of inflammation in atherosclerosis. Both established and novel inflammatory markers have been evaluated in primary and secondary prevention of CVD and are now recognized as promising biomarkers of CVD. For example, the C-reactive protein (CRP), an acute-phase protein produced predominantly by hepatocytes in response to acute infection, inflammatory conditions, and trauma [4] was shown to predict cardiovascular mortality in the general population in a large meta-analysis including 83,995 participants [5]. However, it is not clear whether CRP plays a causal role in the CVD development [6] or is only a marker of subclinical inflammation caused by atherogenic LDL particles. Therefore, other members of the inflammatory signaling cascade have been examined, such as lipopolysaccharide-binding protein (LBP). LBP is a serum acute-phase protein produced by hepatocytes and intestinal epithelial cells, which binds bacterial lipopolysaccharide and thus increases its capacity to induce cytokine release by mononuclear phagocytes [7]. LBP has been associated with coronary artery disease [8] and total and cardiovascular mortality [9].

Arterial calcifications have also been found to be strong predictors of both all-cause and cardiovascular mortality [10]. Coronary artery calcification has been associated with the degree of atherosclerosis and the rate of future cardiac events [11], and it is now accepted as a valuable predictor of coronary heart disease [12]. The important pathological mechanisms are the calcification of tunica intima related to lipid deposits and infiltration of inflammatory cells [13], and calcification of the tunica media resulting from transformation of vascular smooth muscle cells into osteoblast-like cells [14] mediated by various mediators of bone metabolism [15], including osteoprotegerin (OPG) and vitamin D.

OPG exerts its effects on ectopic mineralization by binding to the receptor activator of nuclear factor kappa-B ligand (RANKL), thus inhibiting its effects on bone resorption [16]. Serum OPG levels were previously associated with coronary artery disease and cardiovascular morbidity and mortality (reviewed in [17]). In our earlier cross-sectional study, OPG was associated with the presence and severity of peripheral arterial disease in 165 patients with type 2 diabetes [18].

Vitamin D, together with parathormone, plays an important role in maintaining the balance between calcium and phosphate serum concentrations. Studies investigating possible association between 25-hydroxyvitamin D [25(OH)D] and arterial calcifications have yielded conflicting results (reviewed in [13]). However, large meta-analyses show an association between vitamin D and increased risk of CVD events and mortality [19,20].

Asymmetric dimethylarginine (ADMA) is another potential biomarker of cardiovascular disease that may affect the endothelial function by reducing barrier integrity while increasing inflammatory markers and production of reactive oxygen species [21]. ADMA has been associated with an increased carotid intima-media thickness [22,23,24], cardiovascular disease [25] and mortality in patients admitted with acute myocardial infarction [26].

Despite the amount of published literature, data on the discussed biomarkers as predictors of cardiovascular outcomes and mortality in the high-risk population of patients with type 2 diabetes are controversial. Therefore, we aimed to investigate the association of selected biomarkers relevant for the development of atherosclerosis (CRP, OPG, 25(OH)D, ADMA, and LBP) with cardiovascular events and mortality in patients with T2D during more than 5-year follow-up period.

## 2. Materials and Methods

### 2.1. Study Population

This study included 190 European descent individuals with type 2 diabetes, among which 106 were male. Ethical consent was obtained from the Ethics Committee of Louis Pasteur University Hospital in Košice, Slovakia (No. 2019/EK/5026). Each subject signed informed consent. The patients were originally included to the cross-sectional study examining risk factors for the severity of peripheral artery disease in diabetes [18]; therefore, they had higher than average risk of developing subsequent cardiovascular events. Subjects were selected from those visiting the Diabetes Outpatient Clinic at the 4th Department of Internal Medicine, Louis Pasteur University Hospital, and the Vascular Medicine Ward at the Department of Cardiology, East Slovak Institute for Cardiovascular Disease, Košice, Slovakia, during the period from January 2014 to July 2016. The diagnosis of diabetes mellitus was based on the ADA diagnostic criteria [27] or the use of antidiabetic medication or insulin. Comprehensive medical histories were obtained from each participant, as well as from their medical documentation, which included details like smoking habits, coronary artery disease, previous cardiovascular events (myocardial infarction and cerebral stroke), peripheral artery disease, hypertension, hypercholesterolemia, diabetes duration, and medication use. Peripheral artery disease (PAD) was diagnosed at baseline using ankle-brachial index or toe-brachial index measurements (in patients with mediocalcinosis), or through documented percutaneous transluminal angioplasty (PTA) or peripheral bypass surgery. For this study, we have collected the data on new cardiovascular events or death that occurred between the baseline visit and the study end date of 10 March 2022. The mean follow-up time was 5.6 ± 2.2 (SD) years.

### 2.2. Cardiovascular and Mortality Data Collection

We searched medical records from Louis Pasteur University Hospital and the East Slovakian Institute for Cardiovascular Disease in Košice to identify cases of myocardial infarction, stroke, transient ischemic attack (TIA), and records of performed interventions or surgeries such as percutaneous coronary intervention, coronary bypass surgery, carotid artery angioplasty, PTA, peripheral bypass surgery, and lower limb amputation up until the date of 10 March 2022, which marked the end of the search. Mortality data were extracted from the national mortality database eMortes [28] as of 10 March 2022. The database registers mortality but does not state the specific causes of death.

### 2.3. Laboratory Measurements

Venous blood samples were collected between 8:00 and 10:00 a.m. following an overnight fast and abstinence from medications, tobacco, alcohol, and tea or coffee. After a 15 min rest period in a supine position, venous blood samples were drawn from the antecubital fossa. Glycated hemoglobin (HbA1c) and low-density lipoprotein cholesterol (LDLC) were measured using routine laboratory methods.

The blood samples were then centrifuged, and the resulting sera were divided into aliquots and stored at −70 °C until analysis. Serum OPG and serum high-sensitivity CRP were assessed by an enzyme-linked immunosorbent assay (ELISA) using commercially available kits (Biovendor, Heidelberg, Germany) on the EVOLISTM System analyzer (Bio-Rad Laboratories, Hercules, CA, USA). Serum 25(OH)D (total 25(OH)D2 and 25(OH)D3 levels) and ADMA were measured using their respective ELISA kits from DLD Diagnostika GmbH (Alderhost, Hamburg, Germany). LBP was measured by ELISA kits provided by MyBiosource Inc. (San Diego, CA, USA).

### 2.4. Statistical Analysis

All statistical analyses were performed using IBM SPSS Statistics version 20 (IBM SPSS, Chicago, IL, USA). To obtain comparable effect sizes, we standardized the markers OPG, 25(OH)D, CRP, LBP, and ADMA based on their respective standard deviations (SDs). The primary outcome was defined as any cardiovascular event or death due to any cause. Secondary outcomes were components of the primary outcome. The considered cardiovascular events included myocardial infarction, stroke, TIA, limb amputation, and any vascular intervention (coronary, cerebrovascular, or peripheral).

Data are expressed as mean ± standard deviation (SD) for normally distributed variables or as median (interquartile range) for variables with non-normal distribution. For univariate analyses *t*-test for independent groups, Mann–Whitney test, and χ^2^-test were used where appropriate. Spearman’s test was used for correlation analysis. Multivariable logistic regression models were used to assess the examined markers as predictors of the primary outcome and it’s both components during the follow-up period. The models were adjusted for baseline cardiovascular status (including previous myocardial infarction, previous stroke, or diagnosis of PAD at baseline), and for cardiovascular risk factors such as age, gender, BMI, HbA1c, LDLC, smoking status, and arterial hypertension. Additional adjustment for baseline antidiabetic treatment (insulin, metformin, and SGLT2-inhibitors) and ACE-inhibitor treatment was performed. Associations are reported as odds ratios (OR) with 95% confidence intervals (CI). *p*-value < 0.05 was considered significant. Due to missing values, we have imputed the data for LDLC (28% of all values) and HbA1c (24% of all values) by the method of mean imputation.

## 3. Results

### 3.1. Characteristics of the Study Participants

The current study enrolled a total of 190 participants with type 2 diabetes mellitus, including 106 men and 84 women. The mean age was 64.9 ± 9.4 years, and the mean BMI was 30.3 ± 5.3 kg/m^2^. Prior to their inclusion, 75 (41.4%) participants had a history of coronary artery disease, 29 (15.7%) experienced a stroke, and 100 (52.6%) were diagnosed with peripheral artery disease. Further characteristics of study participants divided according to the presence of primary outcome (incidence of any cardiovascular event or all-cause mortality) are shown in Table 1.

### 3.2. Incidence of Cardiovascular Events

Table S1 provides information on cardiovascular events occurring during the follow-up, which had an average duration of time 5.6 ± 2.2 years. A total of 52 participants (27.4%) experienced a cardiovascular event of some sort. The all-cause mortality rate was 31.1% during the follow-up period. In total, 89 participants (46.8%) either suffered a cardiovascular event or died from any cause during the study. High incidence of cardiovascular events during the follow up period suggest that the study population was at high cardiovascular risk.

### 3.3. Associations of Selected Biomarkers with the Primary Outcome

Table 1 displays a comparison of participants who suffered a new cardiovascular event or died (primary outcome group) and those who survived the follow-up period without a new cardiovascular event (control group). Participants in the primary outcome group were significantly older, had higher mean HbA1c levels, but lower mean BMI. There were also higher proportion of females, smokers, and patients with baseline cardiovascular disease in the primary outcome group. The primary outcome group had significantly lower 25(OH)D levels and significantly higher OPG and CRP levels compared to the control group. There were no significant differences in LBP or ADMA levels between the groups.

We further analyzed separately the associations of investigated biomarkers with the primary outcome during the follow-up. The strongest association with the primary outcome was observed with 25(OH)D in the unadjusted model. This inverse association remained significant in the partially adjusted model for risk factors and baseline CVD (OR 0.41, 95% CI 0.26–0.65) and after full adjustment including baseline antidiabetic and ACE inhibitor treatment (Table 2). CRP and LBP levels were also significantly related to the primary outcome in the fully adjusted model.

We conducted further analysis to test different cut-off values for vitamin D deficiency (<20, <15, <10 ng/mL). Patients with 25(OH)D < 15 ng/mL had significantly increased OR of 2.77 (95% CI 1.14–6.71, *p* = 0.024) in the fully adjusted model for the primary outcome when compared with patients with 25(OH)D ≥ 15 ng/mL (Table S3).

CRP showed a significant positive association with primary outcome in the basic and both adjusted models while LBP only in the adjusted models, and OPG only in the unadjusted model. No significant association of ADMA with primary outcome was observed in both unadjusted and fully adjusted models (Table 2).

### 3.4. Associations of Selected Biomarkers with the Components of Primary Outcome

None of the evaluated biomarkers predicted the incidence of any cardiovascular events in analysis adjusted for baseline CVD and established risk factors. Also, we found no significant relationship between biomarkers levels and specific cardiovascular endpoints (myocardial infarction, stroke, lower limb amputation or any revascularization (data not shown) probably because of low statistical power for such analyses since only a limited number of participants suffered any of the specific outcome included in “any cardiovascular event” category.

The association of biomarkers with all-cause death is shown in Table 3. In unadjusted analysis 25(OH)D, OPG, CRP, and LBP were significantly associated with all-cause death. In the fully adjusted model only the associations of 25(OH)D and CRP with all-cause death remained statistically significant.

Further analysis testing different cut-off values for vitamin D deficiency (<20, <15, <10 ng/mL) showed that patients with 25(OH)D < 10 ng/mL had significantly increased OR of 2.95 (95% CI 1.22–7.13, *p* = 0.016) in the fully adjusted model for the primary outcome when compared with patients with 25(OH)D ≥ 10 ng/mL. The differences in mortality using further two cut-off points were not significant (Table S3).

### 3.5. Multivariable Logistic Regression Analysis

Given the significant correlations among the studied biomarkers (Table S2), we further explored their predictive value for the primary outcome using a multivariable logistic regression analysis with forward stepwise selection of the variables. Among the five studied biomarkers, only 25(OH)D significantly predicted the primary outcome (OR 0.43, 95% CI 0.28–0.66), which corresponds to an inverse OR 2.33 (95% CI 1.51–3.57) for lower baseline 25(OH)D levels by 1 SD (10 ng/mL). Age, female sex, and baseline presence of CVD were also significant predictors of the primary outcome (Table 4). The final model explained 48% of the primary outcome variance.

None of the evaluated biomarkers was a significant predictor of the incidence of any cardiovascular event in a multivariable logistic regression model. Among the established cardiovascular risk factors baseline CVD, HbA1c, and BMI were included in the final model which explained 31% of variance in incidence of cardiovascular events (Table 4).

25(OH)D level was a significant predictor of all-cause mortality in this model (OR 0.50, 95% CI 0.33–0.76) meaning that reduction in 25(OH)D level by ≈10 ng/mL was associated with a double increase in all-cause mortality in our group of patients with diabetes. Further significant predictors in the final model were age and female sex (Table 4). The final model explained 32% of the all-cause mortality variance.

## 4. Discussion

Among novel biomarkers studied 25(OH)D, CRP, and LBP significantly predicted incidence of any cardiovascular event or total mortality in a group of patients with type 2 diabetes during a mean 5.6-year follow-up when analyzed separately. When all studied biomarkers were included in a multivariable model, only decreased level of 25(OH)D remained a significant predictor of the primary composite outcome. In addition, higher age, female sex, and baseline presence of previous cardiovascular events also significantly predicted the incidence of primary outcome.

The effects on the components of primary outcome were different. While none of the examined biomarkers was associated with cardiovascular events in multivariable adjusted models, 25(OH)D and CRP predicted all-cause mortality even after multivariable adjustment. With all studied biomarkers included in the model, only 25(OH)D was associated with all-cause death.

Vitamin D plays a crucial role in regulation of bone and mineral metabolism. In addition, vitamin D receptors are expressed in almost all human cells which suggests a widespread role of vitamin D for overall health via influencing several extra-skeletal organs such as immune system, cardiovascular system, central nervous system, and probably playing a role in defense against developing malignant tumors [29]. Observational studies in humans have shown an inverse relationship between vitamin D levels and cardiovascular events. A meta-analysis of prospective studies including more than 65,000 subjects demonstrated an inverse relationship between 25(OH)D levels and risk for cardiovascular disease with relative risk (RR) of 1.03 (95% CI 1.00–1.60) per 10 ng/mL decrease [30]. Low vitamin D levels were also associated with all-cause mortality according to several studies [20,31,32,33,34]. In the largest meta-analysis including 849,412 study participants from 73 cohort studies with 66,511 mortality events, the RR for mortality, adjusted for potential risk factors, was 1.35 (95% CI 1.22–1.49) in the bottom versus the top third of baseline 25(OH)D levels [31]. The association was also confirmed in various population subgroups such as individuals with diabetes. For example, in a large prospective study of 6329 individuals with diabetes, higher serum 25(OH)D levels were significantly associated with lower all-cause and CVD mortality [32]. Our results are in accordance with those observations for the composite outcome of any cardiovascular event or total mortality. 25(OH)D was the only significant predictor of the primary outcome and total mortality in multivariable analyses including all five studied biomarkers and adjusted for baseline cardiovascular disease and established cardiovascular risk factors. For each SD (10 ng/mL) 25(OH)D decrease, 2.3-times increase in primary outcome and 2-times increase in all-cause mortality was observed. Of note, both the group of patients with primary outcome and the group of patients who did not suffer this outcome had median 25(OH)D levels in the range of vitamin D deficiency (7.8 and 18.2 ng/mL, respectively). Thus, the observed association of 25(OH)D with the primary outcome seemed to be mainly driven by the all-cause mortality rather than incidence of cardiovascular events. We also tested the effect of different cut-off values for vitamin D deficiency in our group of diabetic patients. Patients with 25(OH)D < 15 ng/mL had significantly increased OR of 2.8 for primary outcome. For all-cause mortality we identified the cut-off value of <10 ng/mL to be significantly associated with 3-times increased all-cause mortality.

The mechanistic links between vitamin D deficiency and increased risk of CVD and mortality have been studied extensively. Vitamin D seems to exert pleiotropic cardiovascular effects by activating nuclear vitamin D receptor (VDR) in cardiomyocytes and vascular endothelial cells and by regulating the renin-angiotensin-aldosterone system, adiposity, and energy expenditure [35]. Vitamin D deficiency has been linked to endothelial dysfunction [36], arterial stiffness [37], and left ventricular hypertrophy and cardiac function [38]. The association between the low levels of 25(OH)D and mortality was found not only for all-cause mortality, but also for mortality due to cardiovascular diseases, cancer, respiratory disease, and non-vascular, non-cancer causes in observational studies [20,31,39,40,41,42,43,44,45]. Results from the Mendelian randomization study, comparing the associations of plasma 25(OH)D levels and of genetically determined 25(OH)D levels with cardiovascular and cancer mortality suggested causality regarding the association of vitamin D deficiency and increased cancer mortality but not cardiovascular mortality [46]. Moreover, a meta-analysis of 50 randomized controlled trials demonstrated that supplementation of D vitamin reduced the risk of cancer death by 15% but was not associated with all-cause mortality [47].

We did not have information on the specific causes of deaths in our study, however, our follow-up period overlaps with the coronavirus disease 2019 (COVID-19) pandemic, which has also significantly contributed to the overall mortality rates in Slovakia. Interestingly, a study performed in Slovak population during the COVID-19 outbreak demonstrated that low 25(OH)D levels at the time of admission were independently associated with higher mortality in 357 patients hospitalized with COVID-19 pneumonia [48]; thus, we cannot exclude that COVID-19 at least partially influenced the mortality in our vitamin D deficient group of patients with diabetes.

In accordance with the results of previous studies, OPG was associated with both primary outcome and all-cause death in univariable, but not in multivariable analysis adjusted for established cardiovascular risk factors. In our previous cross-sectional study, we found that OPG was associated with the toe-brachial index as an expression of severity of peripheral artery disease in patients with diabetes. However, this association did not remain significant after introducing 25(OH)D in the multivariable model [18]. Likewise, in a study with elderly women, OPG was a significant predictor of all-cause mortality in univariable and some partially adjusted models, but not in a multivariable adjusted model [49]. That does not exclude the direct pathogenic role of OPG in the progression of atherosclerosis by promoting calcifications of the arterial walls. OPG was in our study strongly inversely correlated with 25(OH)D (r = −0.61, *p* < 0.001) (Table S2). Thus, it is possible that OPG might be one of the mediators of decreased 25(OH)D effect.

CRP, a marker of subclinical inflammation, was also a significant predictor of primary outcome in a multivariable adjusted model. Risk of primary outcome was increased by 87% per SD (4.8 mg/L) of CRP increase. It did not remain significant in the model including all biomarkers, probably because of the collinearity with 25(OH)D levels (r = −0.41, *p* < 0.001) (Table S2). This finding confirmed previous observations reflecting the role of vitamin D in inflammation and reducing the levels of inflammatory markers after vitamin D supplementation [50,51]. Most of the previous evidence points to the hypothesis that CRP is rather a marker of progressing atherosclerosis [52,53] than a causal risk factor for CVD [54]. Our results also support the role of CRP as a marker of cardiovascular disease. Increased CRP levels reflect probably both subclinical inflammation present in patients with type 2 diabetes and decreased 25(OH)D levels.

Analogically with CRP, LBP was also significantly associated with both primary outcome and all-cause mortality in multivariable adjusted models, but not in the final model analyzing all examined biomarkers. LBP is another marker of inflammation, levels of which are increased after previous infections with bacteria such as *Chlamydia pneumoniae*, *Helicobacter pylori*, or *Porphyromonas gingivalis* [55]. In the present study, LBP levels significantly correlated with CRP (r = 0.35, *p* < 0.001) but not with 25(OH)D (Table S2). These results agree with a previous study in 247 men undergoing coronary angiography in whom LBP was a significant predictor of coronary artery disease independent from other established risk factors [8]. We hypothesize that decreased 25(OH)D levels predispose also to infections by different bacteria that subsequently result in increased LBP levels and inflammation that may participate in the process of atherogenesis.

The strength of this study is the detailed characteristics of the study population at baseline with respect to previous CVD and long-term follow-up of 5.6 years in average. We adjusted in statistical analyses for the presence of baseline CVD and all established risk factors. The present study has some limitations. The number of subjects included was relatively low leading to low statistical power of the study to detect small differences. Our study group had higher than average incidence of cardiovascular events by inclusion criteria. Thus, its results cannot be extrapolated to the entire population of patients with type 2 diabetes. From the database of deceased we were not able to identify exact cause of death and subsequently evaluate cardiovascular mortality. We were also not able to identify the percentage of patients that suffered from COVID-19 and, thus, could not exclude that COVID-19 related deaths may have contributed to differences in all-cause mortality.

## 5. Conclusions

The present study showed that vitamin D deficiency was the strongest factor associated with the primary study outcome and all-cause mortality after 5.6-year follow-up in patients with type 2 diabetes at high cardiovascular risk. CRP, LBP, and OPG are probably only markers of ongoing process of atherosclerosis that reflect vitamin D deficiency. Further studies are needed to confirm this hypothesis. However, the present study supports the rationale of vitamin D supplementation in patients with type 2 diabetes at high cardiovascular risk, especially in those with obesity, higher age, and darker skin tone [56].

## Figures and Tables

**Table 1:** Clinical characteristics and marker levels in patients with type 2 diabetes divided according to presence of any cardiovascular event or all-cause death.

	Any Cardiovascular Event or Death (n = 89)	No Cardiovascular Event and Alive (n = 101)	*p*
Age (years)	68.8 ± 8.6	62.2 ± 9.0	<0.001
Sex (% women)	51.5	36.6	0.040
Duration of diabetes	7.1 ± 6.6	12.5 ± 8.5	<0.001
Hypertension (%)	88.5	93.9	0.202
Smoking (%)	-	-	0.033
Never	42.7	61.0	
Previous	18.0	15.0	
Current	39.3	24.0	
CVD at baseline (%)	-	-	<0.001
None	13.6	59.4	
1 vascular bed	46.6	27.1	
2 vascular beds	35.2	11.5	
3 vascular beds	4.5	2.1	
BMI (kg/m^2^)	29.4 ± 5.6	31.0 ± 4.8	0.043
HbA1c (%)	7.93 ± 1.64	7.31 ± 1.67	0.012
LDL cholesterol (mmol/L)	2.61 ± 0.81	2.76 ± 0.74	0.170
Creatinine (µmol/L)	88.9 ± 44.5	78.3 ± 19.6	0.031
*Biomarkers (median, IQR)*			
25(OH)D (ng/mL)	7.82 (1.27–16.7)	18.2 (14.3–24.2)	<0.001
OPG (pmol/L)	19.7 (10.1–27.5)	7.76 (5.83–20.2)	<0.001
CRP (mg/L)	8.03 (2.36–11.6)	3.65 (1.64–7.71)	0.018
LBP (nmol/L)	49.0 (36.9–62.7)	45.8 (27.5–59.7)	0.123
ADMA (µmol/L)	0.40 (0.34–0.53)	0.44 (0.36–0.56)	0.076
*Antidiabetic medication (%)*			
Insulin	48.3	18.8	<0.001
Sulfonylurea	23.3	35.4	0.073
Metformin	47.7	79.2	<0.001
Glitazones	0	1.0	0.347
DPP-4 inhibitors	11.5	18.8	0.173
GLP-1 agonists	1.2	3.1	0.367
SGLT2 inhibitors	0.1	5.2	0.031
*Antihypertensive medication (%)*			
ACE inhibitors	69.2	83.3	0.031
Calcium blockers	37.2	44.0	0.372
Diuretics	39.7	35.2	0.539
Beta-blockers	53.8	58.2	0.566
Centrally acting drugs	20.5	20.9	0.953
*Lipid-lowering medication (%)*			
Statin	65.0	70.1	0.480
Fibrate	8.9	16.1	0.162
Ezetimibe	3.8	2.3	0.573

Data are displayed as arithmetic mean ± standard deviations or percentages or median (interquartile range, IQR). CVD—cardiovascular disease, BMI—body mass index, HbA1c—glycated hemoglobin, LDL—low-density lipoprotein, 25(OH)D—25-hydroxy vitamin D, OPG—osteoprotegerin, CRP—C-reactive protein, LBP—liposaccharide binding protein, ADMA—asymmetric dimethyl arginine.

**Table 2:** Odds ratios of individual markers for composite primary outcome—any cardiovascular event or all-cause death.

	Unadjusted Analysis			Adjusted Analysis			
	OR	95% CI	*p*	OR	95% CI	*p*	*p* *
25(OH)D	0.35	0.24–0.51	<0.001	0.41	0.26–0.65	<0.001	0.005
OPG	1.50	1.02–1.07	<0.001	1.20	0.82–1.76	0.339	0.703
CRP	1.10	1.04–1.18	<0.001	1.87	1.24–2.82	0.003	0.008
LBP	1.32	0.98–1.78	0.064	1.60	1.06–2.42	0.025	0.037
ADMA	0.75	0.55–1.02	0.071	0.96	0.64–1.43	0.831	0.501

Multiple logistic regression model adjusted for the presence of baseline cardiovascular disease, age, sex, BMI, HbA1c, LDL cholesterol, smoking, and hypertension, *p* * additionally adjusted for baseline treatment (insulin, metformin, SGLT2-inhibitors, and ACE-inhibitors). OR (95% CI) are given in SD units. 25(OH)D—25-hydroxy vitamin D, OPG—osteoprotegerin, CRP—C-reactive protein, LBP—liposaccharide binding protein, ADMA—asymmetric dimethyl arginine, OR—odds ratio, CI—confidence interval, SD—standard deviation.

**Table 3:** Odds ratios of individual markers for all-cause mortality.

	Unadjusted Analysis			Adjusted Analysis			
	OR	95% CI	*p*	OR	95% CI	*p*	*p* *
25(OH)D	0.44	0.30–0.64	<0.001	0.56	0.36–0.86	0.008	0.032
OPG	1.80	1.28–2.52	0.001	1.37	0.94–2.01	0.104	0.237
CRP	1.59	1.16–2.19	0.004	1.60	1.24–2.82	0.019	0.035
LBP	1.41	1.02–1.94	0.037	1.51	1.03–2.23	0.036	0.093
ADMA	0.85	0.61–1.18	0.318	1.10	0.68–1.51	0.968	0.604

Multiple logistic regression model adjusted for the presence of baseline cardiovascular disease, age, sex, BMI, HbA1c, LDL cholesterol, smoking, and hypertension, *p* * additionally adjusted for baseline treatment (insulin, metformin, SGLT2-inhibitors, and ACE-inhibitors). OR (95% CI) are given in SD units. 25(OH)D—25-hydroxy vitamin D, OPG—osteoprotegerin, CRP—C-reactive protein, LBP—liposaccharide binding protein, ADMA—asymmetric dimethyl arginine, OR—odds ratio, CI—confidence interval, SD—standard deviation.

**Table 4:** Multivariable models for composite primary outcome and its components with all biomarkers included in the analysis.

Independent Variables	All Cardiovascular Events or All-Cause Death			All Cardiovascular Events			All-Cause Death		
	OR	95% CI	*p*	OR	95% CI	*p*	OR	95% CI	*p*
Age (year)	1.10	1.04–1.15	<0.001				1.12	1.06–1.17	<0.001
CVD baseline	2.58	1.61–4.15	<0.001	3.11	1.93–5.00	<0.001			
25(OH)D (SD)	0.43	0.28–0.66	<0.001				0.50	0.33–0.76	0.001
Sex (men/women)	0.32	0.14–0.71	0.005				0.37	0.17–0.79	0.011
BMI (kg/m^2^)				0.92	0.85–0.99	0.032			
HbA1c (%)				1.38	1.11–1.71	0.004			

Multiple forward stepwise logistic regression models adjusted for the presence of baseline cardiovascular disease, age, sex, BMI, HbA1c, LDL cholesterol, smoking, and hypertension. CVD—cardiovascular disease, 25(OH)D—25-hydroxy vitamin D, SD—standard deviation unit. Empty cells indicate that variable did not enter the final model.

## Data Availability

The data presented in this study are available on request from the corresponding authors. The data are not publicly available because of being clinical data.

## Supplementary Materials

The following supporting information can be downloaded at: https://www.mdpi.com/article/10.3390/metabo13080887/s1, Table S1: Incidence of cardiovascular events and all-cause mortality in 190 participants during the 5.6-follow-up study; Table S2: Spearman’s correlation coefficients between the studied biomarkers; Table S3: Odds ratios of individual categories of 25(OH)D for composite primary outcome and all-cause mortality.
