# Supplementary material for: Decreased 25-Hydroxy Vitamin D Level Is Associated with All-Cause Mortality in Patients with Type 2 Diabetes at High Cardiovascular Risk
Source: Metabolites. 2023 Jul 27;13(8):887. doi: 10.3390/metabo13080887 (PMC10456820; doi:10.3390/metabo13080887)
Supplement: Supplementary file 1 [file metabolites-13-00887-s001.zip › metabolites-2520636-supplementary.pdf]

Supplementary Data

Table S1. Incidence of cardiovascular events and all-cause mortality in 190 participants during the 5.6-year follow-up.

Event	n	%
Any cardiovascular event or death	89	46.8
Any cardiovascular event	52	27.4
Death from any cause	59	31.1
Myocardial infarction	9	4.7
Percutaneous coronary intervention	10	5.3
Coronary bypass surgery	5	2.6
Stroke	10	5.3
Transient ischemic attack	5	2.6
Percutaneous transluminal angioplasty	30	15.8
Peripheral bypass surgery	11	5.8
Lower limb amputation	16	8.4

Table S2. Spearman's correlation coefficients between the studied biomarkers.

	25(OH)D	OPG	hsCRP	LBP	ADMA
25(OH)D	-	-0.610**	-0.405**	-0.140	0.365**
OPG	-0.610**	-	0.343**	0.162*	-0.407**
hsCRP	-0.405**	0.343**	-	0.352**	-0.130
LBP	-0.140	0.162*	0.352**	-	0.020
ADMA	0.365**	-0.407**	-0.130	0.020	-

*P<0.05, **P<0.001; 25(OH)D—25-hydroxy vitamin D, OPG—osteoprotegerin, hsCRP—high sensitivity C-reactive protein, LBP—lipopolysaccharide binding protein, ADMA—asymmetric dimethyl arginine

Table S3. Odds ratios of individual categories of 25(OH)D for composite primary outcome and all-cause mortality.

Categories of		Primary outcome			All-cause mortality	
25(OH)D	OR	95% CI	p	OR	95% CI	p
Cut-off 20 ng/ml	1.84	0.70 - 4.81	0.214	1.58	0.58 - 4.26	0.368
Cut-off 15 ng/ml	2.77	1.14 - 6.71	0.024	1.78	0.74 - 4.27	0.195
Cut-off 10 ng/ml	3.77	1.49 - 9.56	0.005	2.95	1.22 - 7.13	0.016

Multiple logistic regression model adjusted for the presence of baseline cardiovascular disease, age, sex, BMI, HbA1c, LDL cholesterol, smoking, hypertension, and baseline treatment (insulin, metformin, SGLT2-inhibitors, and ACE-inhibitors). Within 190 patients, the "high"category includes 61 patients for cut-off value 20 ng/ml, 96 patients for cut-off value 15 ng/ml, and 124 patients for cut-off value 10 ng/ml. High category is the reference category. 25(OH)D - 25-hydroxy vitamin D, OR – odds ratio, CI –confidence interval.
